# Dense Hydrated Magnesium Carbonate MgCO_3_·3H_2_O Phases

**DOI:** 10.1021/acs.inorgchem.4c01699

**Published:** 2024-08-12

**Authors:** Benedito Donizeti Botan-Neto, David Santamaria-Perez, Lkhamsuren Bayarjargal, Elena Bykova, Javier Gonzalez-Platas, Alberto Otero-de-la-Roza

**Affiliations:** † Departamento de Física Aplicada-ICMUV, MALTA Consolider Team, Universitat de València, Valencia 46100, Spain; ‡ Institute of Geosciences, Goethe University Frankfurt, Frankfurt 60438, Germany; § Departamento Física, Instituto Universitario de Estudios Avanzados en Física Atómica, Molecular y Fotónica (IUDEA), MALTA Consolider Team, Universidad de La Laguna, Tenerife 38204, Spain; ∥ Departamento de Química Física y Analítica, Facultad de Química, MALTA Consolider Team, Universidad de Oviedo, Oviedo 33006, Spain

## Abstract

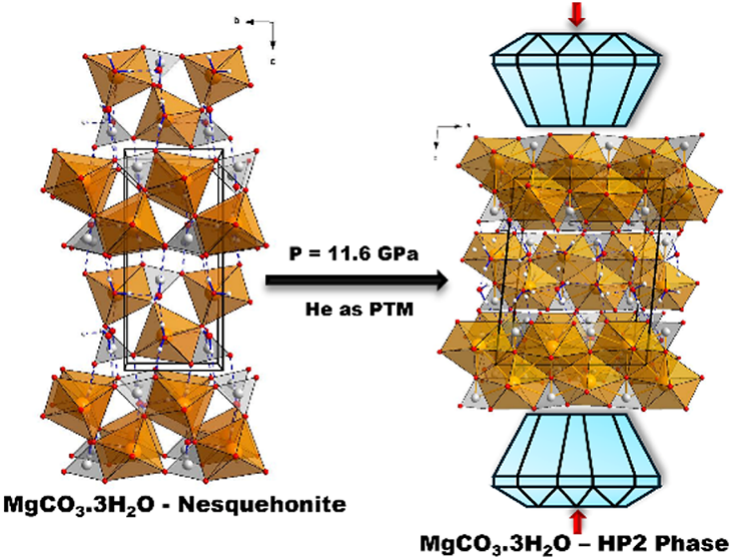

The study of the structural stability of carbonates under different pressure and temperature conditions is important for modeling the carbon budget in the Earth’s interior and the stability of carbonation products of carbon capture and storage (CCS) solutions. In this work, we confirm the existence of the two dense polymorphs of the hydrated magnesium carbonate MgCO_3_·3H_2_O nesquehonite mineral previously reported, and we characterize their structures using synchrotron single-crystal X-ray diffraction at 3.1 and 11.6 GPa. Phase transitions entail the distortion and atomic rearrangement of the Mg-centered polyhedra and the tilting of the [CO_3_] carbonate units. In particular, the Mg coordination number increases from 6 in nesquehonite to 7 in the second high-pressure phase, while maintaining a topology based on complex MgCO_3_(H_2_O)_2_ chains. We also studied their vibrational behavior upon compression using Raman spectroscopy and complemented the experimental results with density-functional theory (DFT) calculations. The role played by hydrogen bonds in the compressibility and the polymorphism of this hydrated carbonate is also discussed.

## Abstract 2

Two novel dense phases of hydrated magnesium carbonate have been structurally and vibrationally characterized by means of in situ synchrotron micron-sized single-crystal X-ray diffraction, Raman spectroscopy, and DFT calculations. Phase transitions entail the increase in the coordination number of Mg atoms, the tilting of the carbonate units, and a higher directionality of H bonds along the less compressible axis. This work gives insight into the stability and the polymorphism in hydrated carbonates.

## Introduction

Increasing concentration of anthropogenic “greenhouse” gases, especially CO_2_, in the Earth’s atmosphere is nowadays considered an important environmental issue. Within the set of strategies for the development of stable carbon dioxide capture and storage (CCS) technologies, a promising approach is its sequestration in a stable form for a long period of time through mineral carbonation.^1,2^ A proper estimation of the stability of this form of CO_2_ storage requires knowledge of the stability of carbonation products at different thermodynamic conditions.^3,4^

Magnesium carbonates exist in a wide variety of naturally occurring minerals.^5−15^ MgCO_3_ magnesite is stable throughout wide pressure and temperature ranges, with only one denser phase known at pressures above 85 GPa and temperatures 2100–2200 K.^16,17^ Magnesite is the thermodynamically stable phase at ambient conditions but, due to strong hydration of the Mg cations in water-rich environments, the anhydrous magnesium carbonate phase is kinetically inhibited at low temperatures^4,18^ and different hydrous and basic carbonates are formed. Nesquehonite is the most commonly observed hydrated magnesium carbonate phase formed by aqueous carbonation reactions at near ambient conditions.^19^ This solid, with formula MgCO_3_·3H_2_O, can be obtained by reacting CO_2_ from industrial gas streams and magnesium from desalination brines in alkaline environments.^20^ Some studies have considered nesquehonite to be a promising permanent and safe solution for CO_2_ storage with potential utilization as a “green” building material, with competitive properties that might be important in a potential industrial approach supporting the circular economy.^20,21^

**Figure 1:**
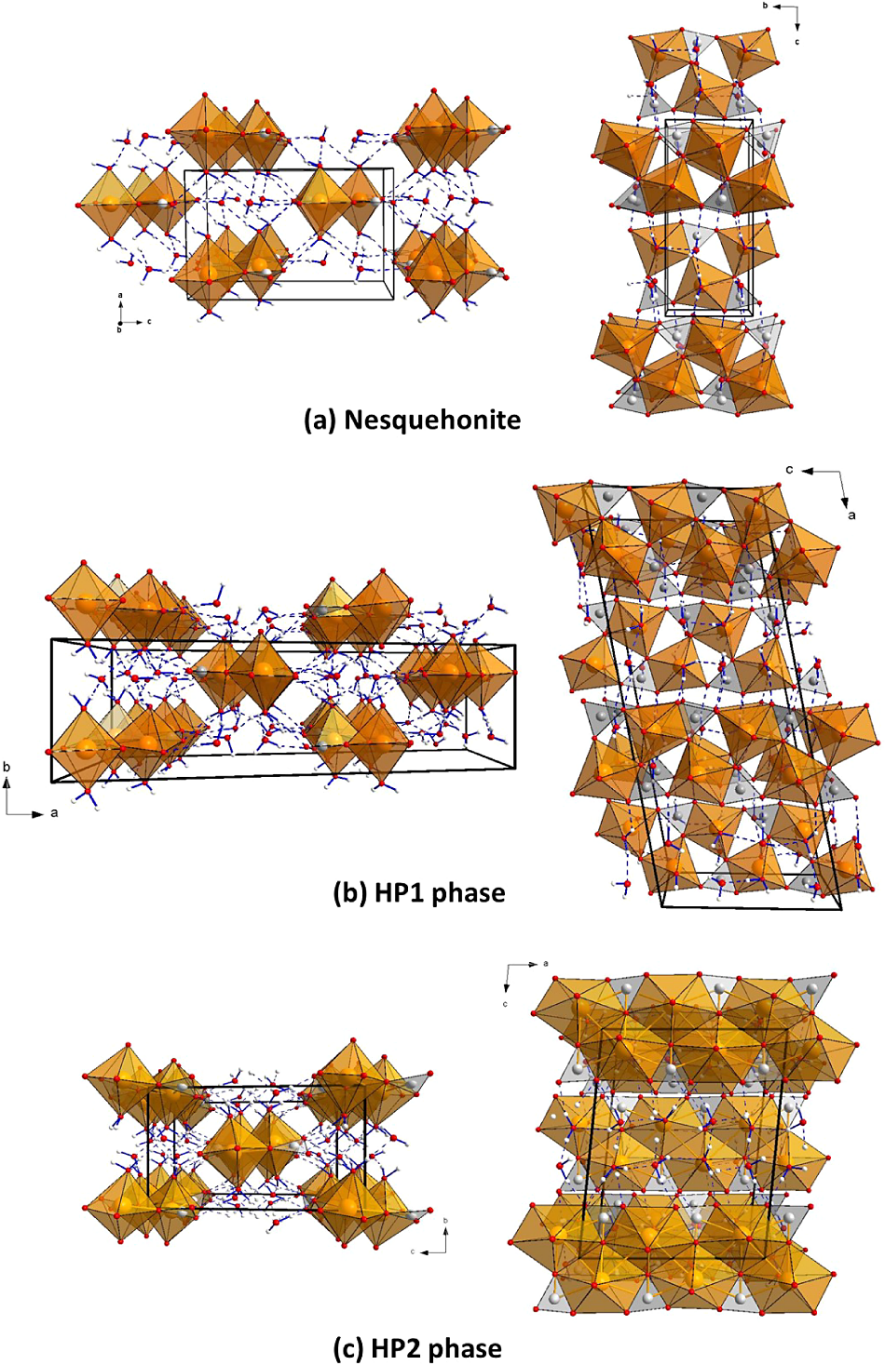
Two projections of the three polymorphs of MgCO_3_·3H_2_O nesquehonite observed upon compression that illustrate the existence and arrangement of complex chains with MgCO_3_·2H_2_O stoichiometry, the interchain water molecules and the H-bonds. (a) Nesquehonite at room conditions perpendicular to the *b* axis (left) and the *a* axis (right). (b) HP1 phase at 3.1 GPa perpendicular to the *c* axis (left) and the *b* axis (right). (c) HP2 phase at 11.6 GPa perpendicular to the *a* axis (left) and the *b* axis (right). Orange, red, gray, and white spheres correspond to the Mg, O, C, and H atoms, respectively. Cation-centered oxygen polyhedra are also depicted. H-bonds are represented as blue dashed lines.

The structure of nesquehonite at ambient conditions has been carefully described in the literature.^7,22−25^ It is characterized by chains along [010], which are formed by strongly distorted corner-sharing [MgO_6_] octahedra, each of them sharing an equatorial edge with a carbonate group and the other two equatorial corners with two other carbonate groups (see Figures 1a and S1). The remaining O atoms completing the octahedral environment belong to two H_2_O ligands. These MgCO_3_(H_2_O)_2_ chains are interconnected only via hydrogen bonds, with one free water molecule situated between the chains. Nesquehonite has been typically reported as a magnesium carbonate with 3 water molecules and a formula unit that can be written as MgCO_3_·3H_2_O.^7,22−25^ However, other studies have suggested the existence of bicarbonate and hydroxyl groups in the structure, and suggest a chemical formula Mg(HCO_3_)(OH)·2H_2_O in nesquehonite specimens formed in acid or neutral conditions (pH < 8).^26,27^

The thermal stability of MgCO_3_·3H_2_O nesquehonite at room pressure is limited to temperatures below 52 °C. Above this temperature loss of weight begins and three dehydratation stages below 250 °C occur, which correspond to the loss of three water molecules per formula unit.^28^ It has been suggested that this decomposition process starts either with the formation of Mg_5_(CO_3_)_4_(OH)_2_·4H_2_O hydromagnesite^28^ or with the formation of an amorphous or ill-crystallized carbonate phase at 115 °C with about two H_2_O molecules in the formula unit.^29^ Recent experiments at 0.7 GPa, alternatively, point to a dehydration with an associated dissociation into a MgCO_3_·4H_2_O phase plus magnesite at the same temperature.^25^ The study of the stability of nesquehonite upon compression at room temperature show the existence of two phase transitions at 2.4(2) and 4.0(3) GPa.^25^ The structure of the first pressure-induced polymorph was only tentatively proposed due to a rapid deterioration of the single-crystals during the XRD experiments. Minor structural differences from nesquehonite seems to come from a slight tilting of [CO_3_] carbonate groups which are no longer fully perpendicular to the *a* axis and produce more irregular [MgO_6_] octahedra (see Figure S2). The structure of the second high-pressure phase was not identified.

In this work we have redetermined the structure of the first dense HP1 phase and determined the structure of the unknown denser HP2 phase after compressing nesquehonite at 3.1 and 11.6 GPa, respectively. The high-pressure polymorphs were characterized in situ by synchrotron microfocus single-crystal X-ray diffraction (XRD) and Raman spectroscopy. Our DFT calculations confirm that the novel HP2 phase is thermodynamically stable at these conditions. A detailed discussion of the atomic arrangements of the structures is provided.

## Experimental Details

Small single crystals of synthetic MgCO_3_·3H_2_O nesquehonite were provided by the Yale Peabody Museum (specimen YPM MIN 031567) from a sample that was prepared by Genth and Penfield more than a century ago.^30^ Chemical analyses were done on a Philips XL30 scanning electron microscope using energy-dispersive X-ray spectroscopy. Only traces (<0.5 at. %) of Mn and Fe were found in addition to the Mg, C, and O atoms present in the ideal MgCO_3_·3H_2_O nesquehonite composition. XRD data confirm the nesquehonite structure,^25^ which has a density (ρ) of 1.83(2) g/cm^3^ at room conditions.

High-pressure experiments were performed in Boehler-Almax-type diamond-anvil cell (DAC) with an 85° aperture angle and diamond culets of 350 μm. A nesquehonite single crystal was loaded into a 150-μm-diameter hole of a rhenium gasket preindented to a thickness of 40 μm. A ruby chip was placed in the chamber to determine the pressure by the fluorescence method.^31^ Helium was loaded in the DAC by means of the Geoscience Institute Frankfurt gas loading apparatus, providing a fluid environment up to 11.6 GPa at room temperature^32^ and a quasi-hydrostatic medium below 30 GPa.^33^

Single-crystal X-ray diffraction was performed at the PETRA III synchrotron (DESY) in Hamburg, Germany, on the Extreme Conditions Beamline P02.2.^34^ The beam was focused by Kirkpatrick Baez mirrors, resulting in a 2.0 (H) × 1.8 (V) μm^2^ (FWHM) spot size on the sample. Diffraction data were collected using a PerkinElmer XRD1621 detector. The wavelength (0.2900 Å) and detector-to-sample distance (401 mm) were calibrated with a CeO_2_ standard using the software DIOPTAS.^35^ The DAC was rotated up to ±35° around the axes perpendicular to the beam, and frames were collected in 0.5° steps with a 4 s acquisition time per frame. The diffractometer and detector geometry were calibrated by measuring a single crystal of enstatite (MgSiO_3_). After the measurement, the reflections were indexed and integrated employing the CrysAlisPRO software.^36^ The subsequent structure solution and refinement were performed using the software packages JANA2006^37^ and Olex2,^38^ employing SHELXT^39^ for the crystal structure determination.

Room-pressure Raman spectroscopic measurements were carried out using a Horiba Jobin Yvon LabRAM HR microspectrometer equipped with a thermoelectrically cooled multichannel charge-coupled device detector and a 1200 grooves/mm grating that allows a high spectral resolution and a 632.8 nm excitation laser. High-pressure Raman spectroscopic measurements were performed with a custom-built setup.^40^ Raman spectroscopy was carried out with an Oxxius LCX-532S Nd:YAG laser (λ = 532.14 nm) in combination with a Princeton Instruments ACTON SpectraPro (SP-2356) spectrograph equipped with a Pixis 256E CCD camera. We used a Raman laser spot size on the sample of ∼6 μm while using a laser power of 26 mW on the sample, in order not to damage the hydrated carbonate compound. The Raman spectra were measured in the range 68–1200 cm^–1^. The Raman region of the OH stretching bands could not be properly measured due to the strong luminescence of the ruby chip.

## Computational Details

The different nesquehonite phases were calculated using density functional theory (DFT) and the projector-augmented wave (PAW) method,^41^ with data sets from the pslibrary^42^ comprising 1 (H), 4 (C), 6 (O), and 10 (Mg) valence electrons. We used the B86bPBE functional^43,44^ with the exchange-hole dipole moment (XDM) model dispersion correction^45−47^ implemented in the Quantum ESPRESSO^48^ suite (version 6.5). The k-point grid for each phase was determined by requiring that a convergence in the total energy of 0.1 mRy and in the pressure of 0.01 GPa. The cutoffs for the plane wave and density expansions were 100 and 1000 Ry, respectively.

The calculation of the equation of state of each phase was done as follows. First, we determined the equilibrium structure at zero pressure and at 50 GPa. Convergence criteria of 10^–4^ Ry/bohr in the maximum force and 10^–5^ Ry in the energy were used. Then, fixed-volume geometry relaxations were carried out on an equally spaced grid of 41 volumes. The equation of state fit as well as the analysis of phase stability as a function of pressure was done using the gibbs2 program.^49,50^ The equations of state for some of the phases show a certain amount of noise, which could be related to the existence of changes under pressure of the optimal placement of the hydrogen bonds. Given that all nesquehonite phases feature extended hydrogen bonding networks, the large number of different proton arrangements and the difficulty in predicting a priori which of them is energetically more favorable, and also the fact that the hydrogen atom barely contributes to the diffraction patterns, we can only use the calculated phase stabilities as a function of pressure as a rough guide for the actual behavior in these systems.

## Results

### Synchrotron Single-Crystal X-ray Diffraction

The present study of nesquehonite upon compression provides new evidence of the existence of two dense polymorphs and determines their structures. At 2.4(2) GPa nesquehonite undergoes a phase transition.^25^ A comparison of synchrotron single-crystal XRD diffraction data at 3.1 GPa (HP1 phase) with previously reported in-home single-crystal diffraction data^25^ revealed the presence of several weak reflections that could have been overlooked in the previous study. The possibility of a different orientation of water molecules in an experimental run using another pressure transmitting medium cannot be ruled out. The diffraction peaks of the synchrotron data set were indexed and integrated. The symmetry of the intensity data set was consistent with Laue group 2/m and the analysis of the systematic absences indicated the centrosymmetric space group *P*2_1_/*c*. At 3.1 GPa, the indexed lattice parameters are *a* = 24.9959(11) Å, *b* = 7.0883(17) Å, *c* = 10.3597(5) Å and β = 101.549(4)° (V = 1798.2(4) Å^3^, Z = 16, ρ_HP1_ = 2.04(3) g/cm^3^). The HKLF 4 data set was used to determine an initial structural model. Half of the hydrogen atoms were located using the residual electron density maps, with their thermal parameters constrained to 1.5 times the U_eq_ value of the neighboring oxygen atoms. The rest of the hydrogen atoms were placed geometrically based on the results from DFT calculations and subsequently refined using soft constraints. The distances between hydrogen and oxygen atoms were constrained to 0.95 ± 0.05 Å, to have distances comparable to those found from density maps. Hydrogen positions were also constrained in order to allow a rotation of the hydrogen around the connected oxygen atom. However, the positions of four hydrogen atoms were not precisely determined, leading to two short H–H interatomic distances (see cif file in the Supporting Information). The weak scattering power of hydrogen atoms and the limited θ range in the diamond anvil cell hinder the precise determination of hydrogen atoms in such a large unit cell. It should be mentioned that the data completeness of our data set was 33% at d = 0.8 Å, far away from 99.5% recommended by the IUCr. Therefore, the description of the experimental hydrogen bond lengths and angles needs to be taken with caution because, even done carefully and under the best possible conditions, data quality is limited.

The structure, solved using the procedure described above, differs only slightly from that previously described in the space group *P*2_1_/*n* with a smaller unit cell: *a* = 7.18(3) Å, *b* = 5.285(2) Å, *c* = 12.116(15) Å and β = 90.1(2)° (V = 459.8(19) Å^3^, Z = 4). The main difference lies in the fact that the previously reported phase possesses four times fewer crystallographically independent atoms, and consequently fewer orientations in carbonate groups and water molecules. It is worth mentioning that attempts to refine the *P*2_1_/*c* model using previously measured single-crystal data at 2.8 and 3.1 GPa^25^ were not successful, which could suggest that several polymorphs with different water orientations could be close in energy and present different local minima in the energy landscape. The details of the XRD data collection, refinement results, and structural data obtained for HP1 single-crystal phase are listed in Tables 1, S1 and S2. The coordination details of the [CO_3_] carbonate groups of the HP1 phase with neighboring [MgO_6_] octahedra and with water molecules through hydrogen bonding are illustrated in Figure S3 (to be compared with those of the previously reported high-pressure structure in Figure S2). LeBail fits using both structural models (Figure 2 of ref (25) and Figure S4) perfectly explain the powder XRD data between 2.4 and 4 GPa and therefore do not allow distinguishing between these models. The pressure dependence of the volume per formula unit obtained using the powder XRD data of ref (25) and the structural model of this study is shown in Figure S5.

**Table 1:** Crystal Data and Structure Refinement for Phase HP1 at 3.1 GPa

CCDC number	2351041
empirical formula	C_4_H_24_Mg_4_O_24_
formula weight	553.4
temperature [K]	293
crystal system	monoclinic
space group (number)	*P*2_1_/*c* (14)
*a* [Å]	24.9959(11)
*b* [Å]	7.0883(17)
*c* [Å]	10.359(5)
α [°]	90
β [°]	101.549(4)
γ [°]	90
volume [Å^3^]	1798.2(4)
*Z*	4
ρ_calc_ [gcm^–3^]	2.0442
μ [mm^–1^]	0.061
*F*(000)	1152
crystal size [mm^3^]	0.001 × 0.001 × 0.001
crystal color	white
crystal shape	submicrometer crystal in a diamond anvil cell
radiation	synchrotron (λ=0.29 Å)
2θ range [°]	3.20 to 36.56
index ranges	–46 ≤ *h* ≤ 39
	–6 ≤ *k* ≤ 6
	–16 ≤ *l* ≤ 17
reflections collected	9947
independent reflections	4026
	*R*_int_ = 0.1053
	*R*_sigma_ = 0.0576
completeness to θ = 13.4°	32.30%
data/restraints/parameters	4026/19/315
goodness-of-fit on *F*^2^	0.869
final *R* indexes [*I* ≥ 2σ(*I*)]	*R*_1_ = 0.0815
	w*R*_2_ = 0.1337
final *R* indexes [all data]	*R*_1_ = 0.2005
	w*R*_2_ = 0.1866
largest peak/hole [eÅ^–3^]	0.21/–0.23

**Figure 2:**
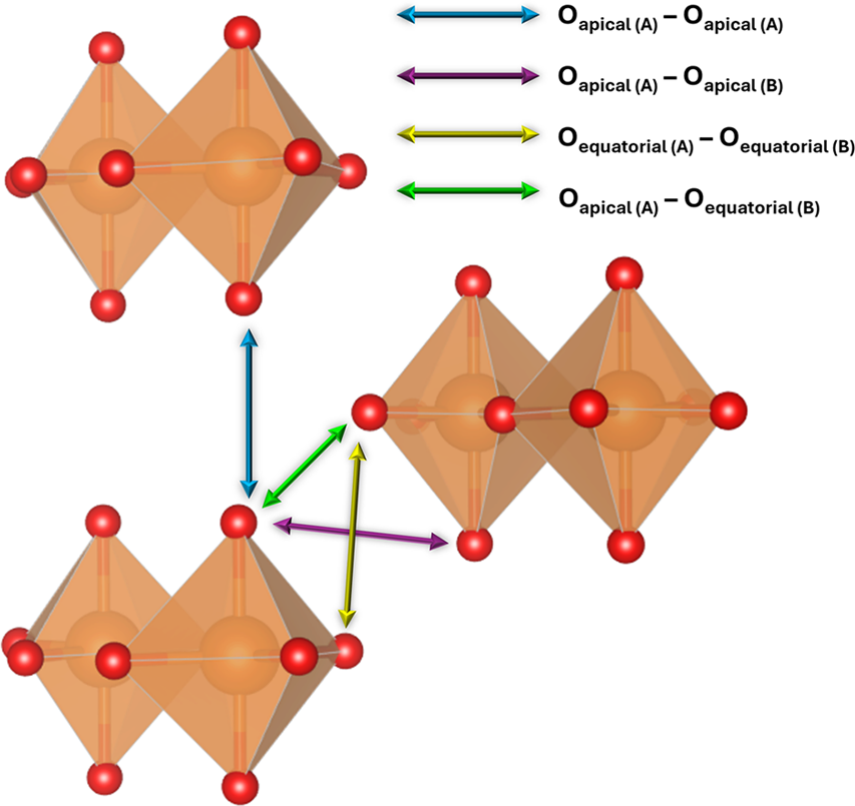
Scheme showing an ideal projection in the direction parallel to the MgCO_3_·2H_2_O chains, with 4 relevant interchain distances that are used in the text to explain their relative displacement upon compression.

The HP1 phase characterized in this study is depicted in Figure 1b and preserves the basic structural topology of the initial nesquehonite (Figure 1a), which consists of double chains of [CO_3_] trigonal and highly distorted [MgO_6_] octahedral groups parallel to the *c* axis (*b* axis in nesquehonite). This strong distortion is due to the fact that [MgO_6_] octahedra and [CO_3_] carbonate groups have a common edge, which explains the very short O–O equatorial distance of the octahedra (∼2.17 Å in comparison with the average of the other O–O equatorial distances, 3.09 Å). The two other equatorial O atoms of the [MgO_6_] units are corners of two [CO_3_] carbonate groups and the apical O atoms belong to two water molecules, forming chains with stoichiometry MgCO_3_·2H_2_O. The remaining H_2_O molecule lies between these double chains connecting them by means of hydrogen bonds. The orientation of the three water molecules and the amount and directionality of the O–H contacts confers the HP1 phase a clear distinction, as discussed later. The carbonate groups, which are roughly parallel to the *bc* plane in nesquehonite, are inclined between 3.17° and 10.56° with respect to the plane *ac* in the HP1 phase (4 independent [CO_3_] groups with tilting angles of 3.17°, 5.52°, 10.34°, and 10.56°), which distort the magnesium octahedra even further. This is evident in the values of the distortion indexes and quadratic elongations of the [MgO_6_] octahedra^51^ which change from 0.01918 and 1.0313 to a mean value of 0.02015 and 1.0341, respectively. The separation between the complex MgCO_3_·2H_2_O chains is also significantly different in the HP1 phase. Note first that a highly anisotropic axial compressibility of the initial nesquehonite structure was previously reported,^25^ the *a* axis being the most compressible axis and the *c* axis slightly expanding upon compression. Additionally, the product of the HP1 phase at 3.1 GPa determined in this study gives a higher value (12.245(1) Å) than the *c* axis of nesquehonite at 2.4 GPa (12.145(2) Å), which means that the larger axis expands at the transition (see Figure 1a,b). This change in the lattice parameters led to a relative displacement of the complex double chains that can be explained in terms of 4 interchain distances (see in Figure 2 the considered distances between A and B chains): O_apical(A)_–O_apical(A)_, O_apical(A)_–O_apical(B)_, O_equatorial(A)_–O_equatorial(B)_, and O_apical(A)_–O_equatorial(B)_. These distances are also affected by the slight corrugation of the chains caused by the tilting of the carbonate groups. Looking at the shortest O–O interchain distances of each type collected in Table 2, it can be clearly inferred that the A (and B) chains closely approach each other in the vertical direction (O_apical(A)_–O_apical(A)_ and O_equatorial(A)_–O_equatorial(B)_ shorter distances decrease considerably) whereas the A and B chains separate in the horizontal direction (O_apical(A)_–O_equatorial(B)_ shorter distance slightly decreases and O_apical(A)_–O_apical(B)_ shorter distance increase). This coordinated displacement between chains leads to or is caused by an increased number of highly directional hydrogen bonds along the long axis.

**Table 2:** Interchain O–O Shortest Distances

	nesquehonite room pressure	HP1 phase 3.1 GPa	HP2 phase 11.6 GPa
O_apical(A)_–O_apical(A)_	3.543 Å	3.125 Å	2.912 Å
O_apical(A)_–O_equatorial(B)_	2.758 Å	2.679 Å	2.647 Å
O_apical(A)_–O_apical(B)_	3.763 Å	3.776 Å	4.649 Å
O_equatorial(A)_–O_equatorial(B)_	4.169 Å	3.438 Å	2.919 Å

A hydrogen bond is a noncovalent interaction, typically depicted as D–H···A and characterized by an angle formed between the ∠DHA atoms, where D–H represents the donor group and A the acceptor. Broadly defined, a hydrogen bond entails an attractive interaction that occurs when the donor group exhibits mild polarity.^52−57^Figure S6 shows a scheme of a hydrogen bond, illustrating its bond length and angle. In this HP1 polymorph, the average H···O length measures 1.90(3) Å, accompanied by an average ∠DHA angle of 155(3)°. The structure relaxed using DFT has hydrogen atoms close to those in the experimental solution. Table 3 collects the average experimental and calculated hydrogen bond lengths (d) and angles (θ) for the different polymorphs of MgCO_3_·3H_2_O, for the sake of comparison. More detailed information on H-bonds of initial nesquehonite and the HP1 phase can be found in Tables S3 and S4, respectively. The reader must keep in mind that experimental hydrogen bond lengths and angles need to be taken with caution because, even with data gathered carefully and under the best possible conditions, data quality is limited (see details of the HP1 and HP2 structure solutions).

**Table 3:** Average Hydrogen Bond Lengths and Angles of the 3 MgCO_3_·3H_2_O Polymorphs at Selected Pressures from Experimental and DFT Calculated Data

	experimental data			DFT calculations		
	pressure (GPa)	H···O (Å)	∠DHA angle (deg)	pressure (GPa)	H···O (Å)	∠DHA angle (deg)
**nesquehonite**	0.0001	1.956	170(2)	0.2460	1.760	164.9
**HP1 phase**	3.1	1.90(3)	155(3)	3.5415	1.711	163.8
**HP2 phase**	11.6	1.76(5)	160(7)	11.7435	1.626	168.3

The second high-pressure polymorph (named HP2 phase), indexed and solved at 11.6 GPa, is described with the *I*2/*a* space group and has the following lattice parameters: *a* = 9.4473(8) Å, *b* = 6.748(5) Å, *c* = 11.8426(11) Å, and β = 95.703(6)° (V = 751.2(6) Å^3^, Z = 8, ρ_HP2_ = 2.45(8) g/cm^3^). The sample, after the phase transition, consisted of two crystalline grains rotated by approximately 180° around the a* axis, as identified through careful inspection of reciprocal space reconstructions. Due to significant overlap (∼22% of all reflections) between the grains, both twin domains were integrated simultaneously using a dedicated twin data processing procedure implemented in the CrysAlisPro software. The detwinned HKLF 4 data set for the first twin domain was used to determine an initial structural model. After solving the structure, most atoms were identified, with the remaining atoms located through a series of difference Fourier map cycles. To enhance the data-to-parameter ratio, the crystal structure refinement was performed using an HKLF 5 data set, which includes intensities from both twin domains. The final twin fraction, refined along with the structure, was 0.572(3). The refinement details of this structural determination are collected in Table 4 and the fractional coordinates and anisotropic displacement parameters are given in Tables S5 and S6, respectively. The crystal structure was refined in anisotropic approximation for all non-hydrogen atoms. Hydrogen atoms were located from the residual electron density maps, with their thermal parameters constrained to 1.2 times the *U*_eq_ value of the oxygen atoms they are bonded to. Soft restraints were applied to the geometry of water molecules, using as a starting model results of DFT calculations, i.e., O–H bond lengths of 0.96 ± 0.05 Å and H–O–H angles of 104.5° (corresponding to a restraint of 1.35 ± 0.1 Å for intramolecular H···H distances). At the same time the H···O hydrogen bond distances were not restrained, allowing water molecule to rotate, which resulted in larger H···O distances (by up to 0.3 Å) compared to theoretical values (see Table S7).

**Table 4:** Crystal Data and Structure Refinement for Phase HP2 at 11.6 GPa

CCDC number	2350918
empirical formula	CH_6_MgO_6_
formula weight	138.37
temperature [K]	293
crystal system	monoclinic
space group (number)	*I2/a* (15)
*a* [Å]	9.4473(8)
*b* [Å]	6.748(5)
*c* [Å]	11.8426(11)
α [°]	90
β [°]	95.703(6)
γ [°]	90
volume [Å^3^]	751.2(6)
*Z*	8
ρ_calc_ [g cm^–3^]	2.447
μ [mm^–1^]	0.069
*F*(000)	576.0
crystal size [mm^3^]	0.001 × 0.001 × 0.001
crystal color	white
crystal shape	submicrometer crystal in a diamond anvil cell
radiation	synchrotron (λ = 0.290 Å)
2θ range [°]	3.536 to 36.588
index ranges	–15 ≤ *h* ≤ 16
	–5 ≤ *k* ≤ 6
	–21 ≤ *l* ≤ 19
reflections collected	1485
independent reflections	1485
	*R*_int_ = 0.0296
	*R*_sigma_ = 0.0369
data/restraints/parameters	1485/60/93
goodness-of-fit on *F*^2^	1.076
final *R* indexes [*I* ≥ 2σ(*I*)]	*R*_1_ = 0.0536
	w*R*_2_ = 0.1434
final *R* indexes [all data]	*R*_1_ = 0.0837
	w*R*_2_ = 0.1614
largest peak/hole [eÅ^–3^]	0.39/–0.38

The HP2 structure is depicted in Figure 1c and is also formed by chains with stoichiometry MgCO_3_·2H_2_O and a “floating” water molecule in the interchain space, as in the initial nesquehonite and the HP1 phases. However, this second pressure-induced transition involves an increase in the coordination of Mg atoms by O atoms from 6 to 7, giving rise to the formation of distorted [MgO_7_] pentagonal bipyramids with an average Mg–O bond length of 2.1093 Å in HP2 phase. Note that this average distance is longer than that observed in [MgO_6_] octahedra of the HP1 phase at a much lower pressure (average Mg–O distance of 2.043 Å at 3.1 GPa). The carbonate groups are bonded to [MgO_7_] units sharing, unlike the HP1 phase, two of their edges with equatorial positions of the pentagonal bipyramid. This results in two short C–O distances of 1.259(4) Å and a larger distance of 1.281(4) Å. The carbonate groups are more tilted than those in HP1 phase with respect to the equatorial plane of the chains, with an inclination angle of 21.95° (see Figures 1c and S7). The average H···O length in this polymorph is 1.76(5) Å, with an average ∠DHA angle of 160(7)°. The hydrogen bonds again play a crucial role in linking the polyhedral units of different chains and interchain water molecules together. Information on H-bonds of the HP2 phase can be found in Table S7. As mentioned above, the structural analysis of the approximation of chains can be conducted by examining the shortest distance O–O between adjacent polyhedra of different chains (see Table 2). From these data, it can be inferred that confronted A or B chains decrease their separation whereas they are positioned to have a greater lateral overlap upon compression. These modifications are related with the higher directionality of the H-bonds along the *c* axis of the HP2 phase. All in all, the transformations result in denser hydrated magnesium carbonate structures.

**Figure 3:**
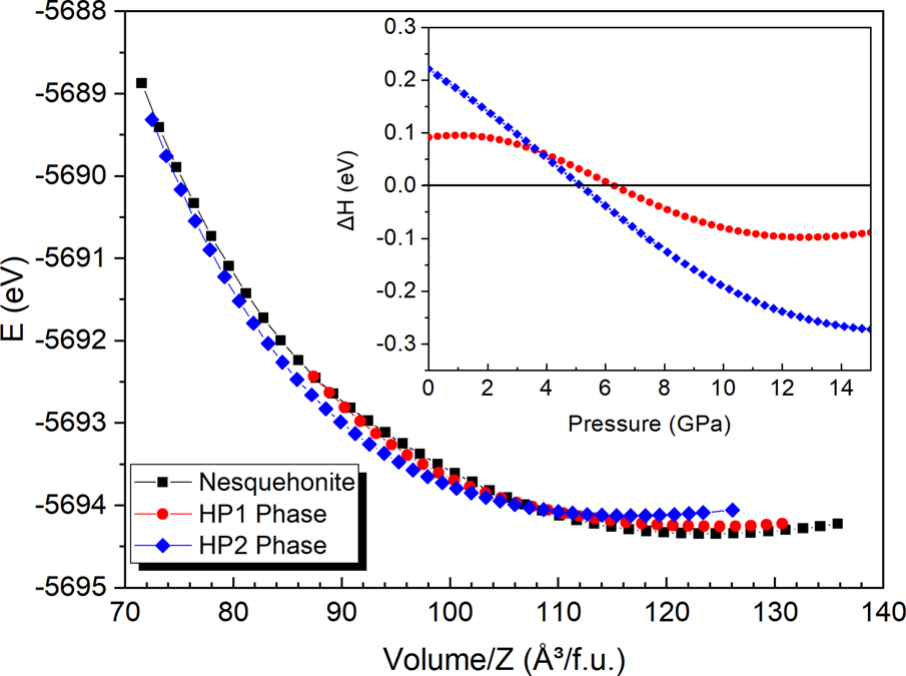
Internal energy as a function of volume per unit cell for the initial P2_1_/n nesquehonite (black), the *P*2_1_/*c* HP1 (red), and the I2/a HP2 (blue) MgCO_3_·3H_2_O phases. The enthalpy variation versus pressure curve for these three polymorphs is depicted in the inset (taking the nesquehonite structure as reference).

DFT calculations confirm that this HP2 phase is the thermodynamically stable structure at high-pressure. Figure 3 shows the energy as a function of volume curves for the initial nesquehonite and the high-pressure HP1 and HP2 calculated structures. The enthalpy vs pressure curves for each phase, referred to the enthalpy of nesquehonite, are shown in the inset. According to our B86bPBE-XdM calculations, nesquehonite is the most stable phase below 5 GPa, the pressure at which the denser HP2 phase becomes more stable, in excellent agreement with the experimental data. On the other hand, the enthalpy curve of the HP1 phase is slightly above that of nesquehonite (∼0.1 eV per formula unit), which suggests that the HP1 phase is an intermediate metastable structure in the transition path to the thermodynamically stable dense HP2 phase. Structural data from DFT calculations are presented in the Supporting Information. The calculated compression dependence of the lattice parameters and unit-cell volumes of the HP2 phase are in good agreement with previously reported experimental data,^25^ showing a smooth and monotonous decrease under pressure. The strong anisotropy of the HP2 postnesquehonite phase is described by the experimental (theoretical) axial compressibility values: β_a_ = 8.5 × 10^–3^ (4.7 × 10^–3^) GPa^–1^, β_b_ = 5.7 × 10^–3^ (5.1 × 10^–3^) GPa^–1^ and β_c_ = 5.0 × 10^–4^ (1.3 × 10^–3^) GPa^–1^, for the *a*, *b*, and *c* axes, respectively. These results indicate that the *c* axis is appreciably less compressible and that the bulk compressibility is dominated by that of the *a* and *b* axes. The experimental (calculated) Birch–Murnaghan equation of state of the HP2 phase gives a bulk modulus B_0_ = 25.2(12) GPa (30.0(2) GPa) when the first-pressure derivative is fixed to the theoretical value B'_0_ = 6.

DFT calculations have also been performed to evaluate the energetics of the MgCO_3_·3H_2_O ⇌ MgO + 2H_2_O + H_2_CO_3_ reaction at high pressures. Benz et al. recently reported the structure of the carbonic acid, H_2_CO_3_, at high pressures.^58^ We carried out calculations for MgO (B1 phase), ice (all phases in ref (59)), and H_2_CO_3_. The equations of state were calculated in the 0 to 30 GPa pressure range, resulting in the ΔH vs P diagram relative to the nesquehonite phase plotted in Figure S8. Our DFT data confirm that the formation of carbonic acid is energetically unfavorable, since the combination of the right-hand side of the above reaction is more than 0.2 eV higher in enthalpy (per formula unit) than the MgCO_3_·3H_2_O polymorphs in the entire pressure range.

### Raman Spectroscopy Measurements

Carbonate Raman spectra can be compared because they contain distinct peaks and bands that vary depending on the mineral’s cation composition and crystal structure.^60^ The Raman spectra regions can be divided into three frequency ranges associated with [CO_3_]^2–^ internal vibrations and [CO_3_] lattice vibrations to the cation. The 600–1200 cm^–1^ region represents the symmetric stretching vibrations of the carbonate group. The asymmetric stretching vibrations of the same group are located in the region 1200–1700 cm^–1^. The lattice vibration region extends from 100 to 500 cm^–1^ and can be divided into a subregion of translational modes (T) below 220 cm^–1^ and a subregion for librational modes (L) above this wavenumber.^61,62^

The Raman spectra of nesquehonite exhibit characteristic peaks in the previously described regions. The symmetry analysis of nesquehonite reveals 168 vibrational modes at the Brillouin zone center (Γ). The mechanical decomposition of these modes is as follows: Γ = 42A_g_(R) + 42B_g_(R) + 41A_u_(IR) + 40B_u_(IR) + A_u_ + 2B_u_, which include 165 optical modes (84 active Raman (R) modes and 81 active infrared (IR) modes) and three acoustic modes.^63^ According to the literature, the most intense Raman peak of nesquehonite is observed around 1100 cm^–1^, corresponding to the symmetric stretching vibration (ν_1_) of the carbonate group.^64,65^ In terms of lattice vibrations, nesquehonite displays peaks at 119, 167, 187, 199, 228, 273, 311, and 344 cm^–1^.^26,57^ Very weak peaks around 707 and 773 cm^–1^ correspond to the in-plane bending mode (ν_4_) of the carbonate group,^27,62,66^ Additionally, weak peaks are observed at approximately 1425 and 1516 cm^–1^, corresponding to the antisymmetric stretching vibration (ν_3_) of the [CO_3_] group. Peaks observed between at 3100 and 3600 cm^–1^ correspond to the OH stretching bands of water molecule vibrations.^65^Table S8 collects the frequencies of the Raman active modes at room conditions from our measurement, which are in good agreement with previous results.

**Figure 4:**
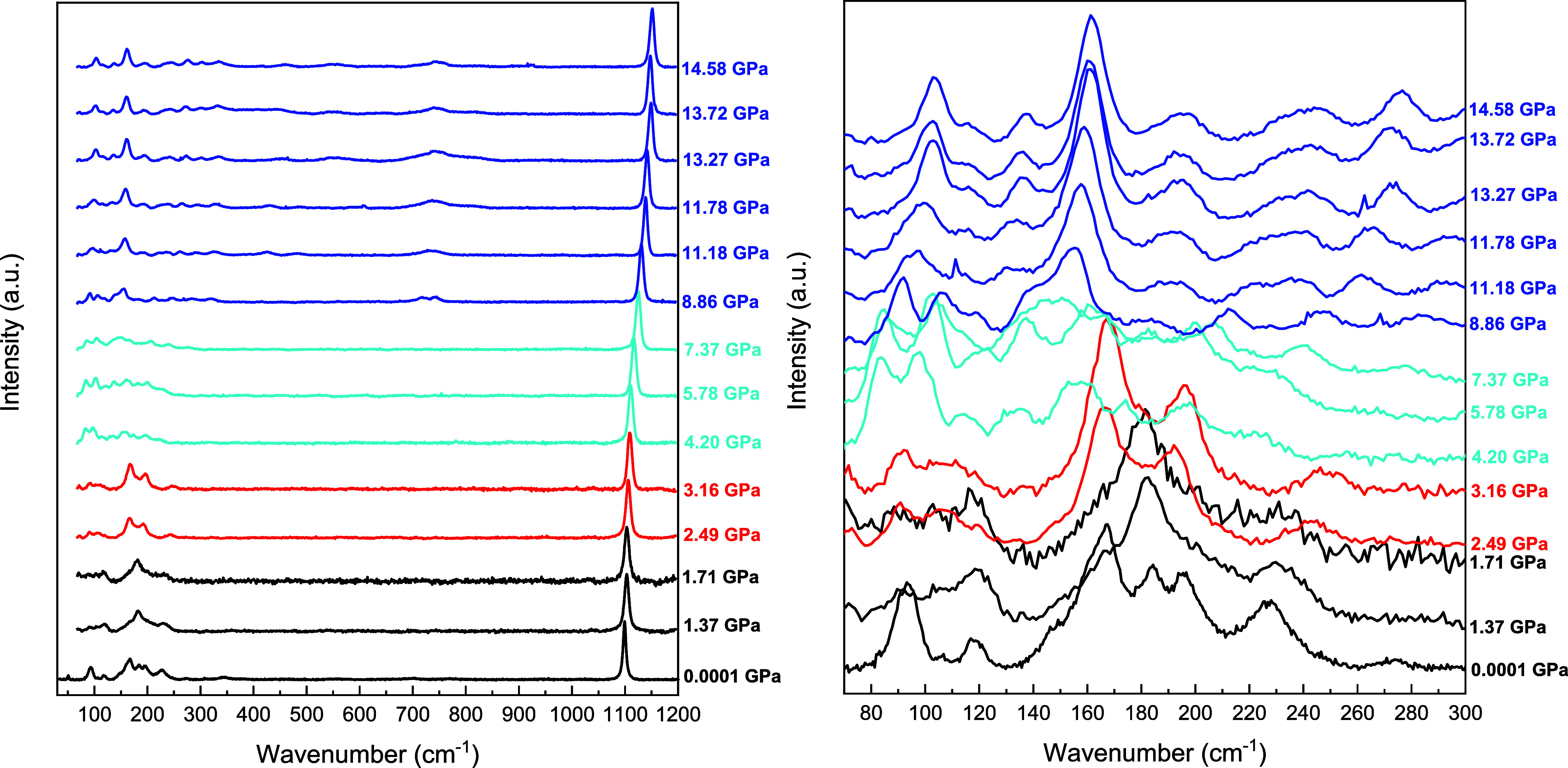
(Left) Room-temperature Raman spectra of MgCO_3_·3H_2_O nesquehonite at selected pressures on upstroke. (Right) Zoom of the 70–300 cm^–1^ wavenumber region.

We carried out high-pressure Raman spectroscopy measurements in nesquehonite up to 17 GPa. Raman spectra at selected pressures are shown in Figure 4. Our Raman data points to the existence of two phase transitions close to 2.5 and 4.1 GPa, with a region of instability in the sample’s local vibrations between 4.1 and 7.8 GPa. In this region, in addition to the HP2 modes, a HP1 Raman mode and an additional mode at ∼160 cm^–1^ can be observed. XRD measurements at 5.3 GPa, on the other hand, show that the sample has already adopted the HP2 phase (see Figure S9), which in principle rules out the existence of any intermediate phase at this pressure. Raman measurements performed in a different experimental run do not exclude a certain degree of heterogeneity in the sample in the transition pressure range. Thus, the onset of the phase transitions obtained using both characterization techniques is in good agreement,^25^ but Raman spectroscopy shows a pressure range (4.1–7.8 GPa) where a continuous transformation into HP2 takes place. The structural transformations occurring below 5.7 GPa mainly entail changes in the intensity of the lattice vibration bands below 260 cm^–1^. The Raman spectra at 5.7 GPa show the onset of the appearance of additional features in the frequency region 600–800 cm^–1^. Above 7.8 GPa, at least 4 new Raman bands clearly emerge in the region 260–430 cm^–1^ and an intense band is clearly visible at 720 cm^–1^, which can be assigned to a carbonate in-plane bending vibration. The increase of intensity of this band seems to be related to the fact that the [CO_3_] group in the HP2 phase shares 2 edges with the [MgO_7_] pentagonal bipyramids.

**Figure 5:**
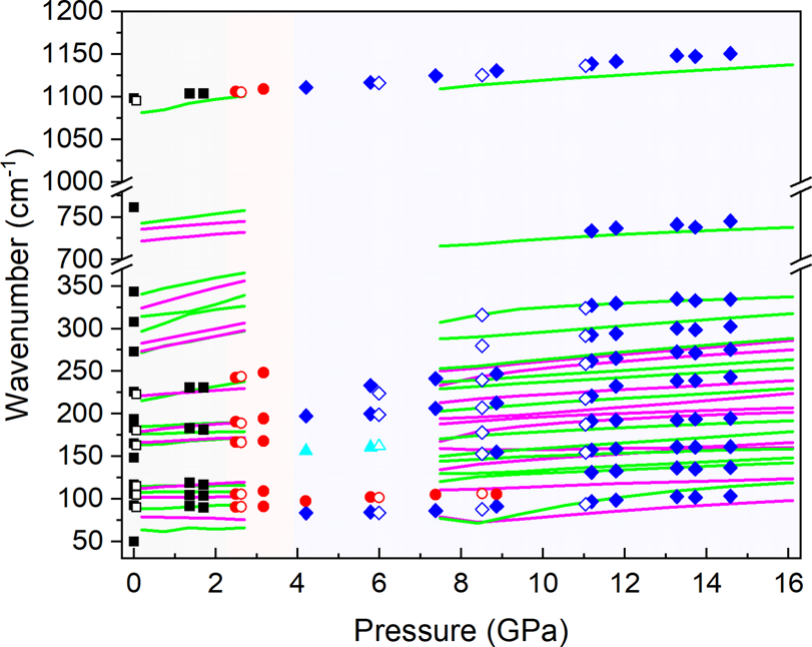
Pressure dependence of the experimental (symbols) and theoretical (lines) Raman-active frequencies of MgCO_3_·3H_2_O. Upstroke and downstroke data are depicted as solid and empty symbols, respectively. Black, red, and blue colors represent the nesquehonite, HP1, and HP2 phases, respectively. Cyan symbols correspond to a mode that likely originates from an instability in the sample’s local vibrations during the HP1–HP2 transformation. Error bars are smaller than the symbol size of each experimental point. The A_g_ and B_g_ DFT-calculated modes are depicted in the graphic as solid green and magenta lines. The DFT calculation modes depicted in the graphic correspond only to the modes observed in the experimental data.

The Raman investigation focuses on tracking the frequency shift of vibrational modes in our sample as pressure varies. Figure 5 displays the experimental upstroke data as solid symbols. Our simulations also provided frequencies and pressure coefficients of the Raman-active optical vibrations of nesquehonite and the HP2 phase (both polymorphs have the same amount of Raman modes according to symmetry analysis). The unit cell of the HP1 phase is too large to compute and obtain valuable information (336 active Raman modes). Notably, the mode of symmetric stretching vibration (ν_1_) located at ∼1100 cm^–1^ is present in all observed structures and shifts smoothly to higher frequencies. The pressure coefficient of the experimentally observed Raman-active frequencies (dω/dP) of this mode above 1.4 GPa is ∼3.8 cm^–1^/GPa. This value is similar to that estimated from DFT calculations of the HP2 phase (3.1 cm^–1^/GPa), but considerably smaller than that estimated for initial nesquehonite (8.2 cm^–1^/GPa). Notably, the four lattice vibration modes associated with Mg atoms observed below 185 cm^–1^ in our nesquehonite Raman spectra below 2 GPa soften with pressure and the structure undergoes the phase transition to the HP1 phase above that pressure. Once the transition takes place the lattice modes slightly harden with increasing pressure. The vibration modes observed at approximately 292 and 327 cm^–1^ (see Figure 5) according to DFT calculations, correspond to L modes, which are exclusively present above 8.5 GPa. Despite the low intensities of these peaks, it seems clear that they represent distinct modes of the HP2 phase. Additionally, at 730 cm^–1^, a vibration mode associated with the in-plane bending vibration (ν_4_) of the carbonate group is clearly identifiable above this pressure in our Raman spectra.

Downstroke measurements are illustrated in the graph using empty symbols. The behavior of most vibrations resembles that observed in the upstroke data. Specifically, at 0.06 GPa, the recovered sample displays the nesquehonite vibration modes plus a residual HP1 vibration mode at 162 cm^–1^. Therefore, as stated in a previous work, the structural behavior of nesquehonite is reversible after decompression.^25^

## Conclusions

This study reports the structural characterization of two dense polymorphs of trihydrated magnesium carbonate MgCO_3_·3H_2_O at 3.1 and 11.6 GPa, named HP1 and HP2 phases, respectively. High-pressure single-crystal XRD measurements using He as hydrostatic pressure medium confirm first the transition of nesquehonite into the structurally distorted HP1 phase, where the complex MgCO_3_·2H_2_O double chains approach apically and the [CO_3_] carbonate groups are no longer coplanar. This structure requires a unit cell four times larger than nesquehonite. The second pressure-induced phase transition entails an increase in the coordination number of the Mg atoms from 6 to 7 oxygen atoms due to the fact that the [CO_3_] carbonate group shares two edges with the adjacent Mg-centered polyhedra, instead of one shared in nesquehonite and the HP1 phase. The apical approximation of the chains causes a higher directionality of the H-bonds along the longer axis. Additionally, Raman spectroscopy measurements show the sequence of phase transitions observed in XRD experiments and illustrate the appearance of distinctive vibrational bands associated with the different coordination environment of the Mg atoms and the different connections between [CO_3_] and [MgO_7_] units observed in the HP2 phase. DFT calculations confirm that the HP2 phase is the thermodynamically stable phase above 5 GPa and the predicted Raman modes explain those experimentally observed. In summary, this work gives insight into the nature of phase transitions of hydrated carbonates upon room temperature compression and reports two novel dense polymorphs.

## Supporting Information Available

Additional tables and figures with structural details of the 3 reported MgCO_3_·3H_2_O phases (PDF)
