# Correction to “Dense Hydrated Magnesium Carbonate MgCO_3_·3H_2_O Phases”

**DOI:** 10.1021/acs.inorgchem.5c00431

**Published:** 2025-02-12

**Authors:** Benedito Donizeti Botan-Neto, David Santamaria-Perez, Lkhamsuren Bayarjargal, Elena Bykova, Javier Gonzalez-Platas, Alberto Otero-dela-Roza

The reference of one funding project was incorrectly indicated.

In the first paragraph of the Acknowledgments “PGC2021-125518NB-I00” should be “PID2021-125518NB-I00”.

