# Supplementary material for: Dense Hydrated Magnesium Carbonate MgCO3·3H2O Phases
Source: Inorg Chem. 2024 Aug 12;63(34):15762–71. doi: 10.1021/acs.inorgchem.4c01699 (PMC11351053; doi:10.1021/acs.inorgchem.4c01699)

Supplementary Material of

Dense hydrated magnesium carbonate $MgCO_3·3H_2O$ phases

Benedito Donizeti Botan-Neto[1], David Santamaria-Perez[1,*], Lkhamsuren Bayarjargal[2], Elena Bykova[2], Javier Gonzalez-Platas[3], Alberto Otero-de-la-Roza[4]

[1] *Departamento de Física Aplicada-ICMUV, MALTA Consolider Team, Universitat de València, Valencia 46100, Spain*

[2] *Institute of Geosciences, Goethe University Frankfurt, Frankfurt 60438, Germany*

[3] *Departamento Física. Instituto Universitario de Estudios Avanzados en Física Atómica, Molecular y Fotónica (IUDEA). MALTA Consolider Team. Universidad de La Laguna, 38204, Tenerife, Spain.*

[4] *Departamento de Química Física y Analítica, Facultad de Química, MALTA Consolider Team, Universidad de Oviedo, Oviedo 33006, Spain*

*Corresponding author e-mail: David.Santamaria@uv.es

Table S1. Atomic coordinates and U_{eq} [Å^2] for the HP1 phase

Atom	*x*	*y*	*z*	U_{eq}
Mg1	0.92354(5)	0.7625(5)	0.90769(10)	0.0154(12)
Mg2	0.82665(5)	0.7322(5)	0.61568(10)	0.0178(12)
Mg3	0.57674(5)	0.2703(5)	0.35194(10)	0.0170(12)
Mg4	0.67343(4)	0.2592(5)	0.65329(10)	0.0156(12)
O1	0.84568(9)	0.7493(9)	0.8212(2)	0.023(2)
O2	0.90445(9)	0.7455(9)	0.5960(2)	0.020(2)
O3	0.76106(9)	0.7459(9)	0.7165(2)	0.023(2)
O4	0.65416(10)	0.2703(10)	0.4467(2)	0.022(2)
O5	0.72118(10)	0.2738(10)	0.3330(2)	0.024(3)
O6	0.59526(9)	0.2550(9)	0.6650(2)	0.023(2)
O7	0.77943(10)	0.7966(9)	0.9345(2)	0.023(3)
O8	0.98843(9)	0.7254(9)	0.5724(2)	0.026(2)
O9	0.52999(10)	0.3058(10)	0.4874(2)	0.034(3)
O10	0.73854(10)	0.2860(9)	0.5534(2)	0.023(2)
O11	0.51124(10)	0.2604(9)	0.6874(2)	0.022(2)
O12	0.97188(10)	0.7536(10)	0.7737(2)	0.027(2)
O13	0.91697(14)	0.0435(14)	0.8817(3)	0.034(4)
H	0.94384	0.10582	0.89281	0.050
C1	0.54541(12)	0.2725(14)	0.6098(3)	0.026(3)
O14	0.82059(14)	0.0179(14)	0.5946(3)	0.049(4)
H14A	0.82567	0.05438	0.51633	0.074
H14B	0.84713	0.07638	0.64887	0.074
O15	0.83368(12)	0.4484(13)	0.6320(3)	0.031(3)
H15A	0.81130	0.39148	0.57221	0.046
H15B	0.86482	0.40978	0.62279	0.046
O16	0.67523(15)	0.9759(14)	0.6426(3)	0.033(4)
H16	0.70277	0.92982	0.69923	0.049
HA	0.67061	0.61154	0.08475	0.049
O17	0.58125(13)	0.9431(12)	0.8287(3)	0.029(3)
H17	0.61298	0.91131	0.81517	0.044
HB	0.56049	0.63641	0.33951	0.044
O18	0.57338(14)	0.9877(13)	0.3748(3)	0.029(4)
H18	0.58605	0.95688	0.45627	0.043
HC	0.54336	0.58276	0.86364	0.043
O19	0.92166(12)	0.2208(11)	0.6340(3)	0.025(3)
H19A	0.95419	0.25912	0.66298	0.038
H19B	0.91487	0.14935	0.69474	0.038
O20	0.92826(14)	0.4812(15)	0.9114(3)	0.049(4)
H20A	0.92587	0.40953	0.99448	0.073
H20B	0.94741	0.37606	0.89260	0.073
C2	0.95510(13)	0.7393(13)	0.6509(3)	0.017(3)
O21	0.67300(13)	0.7633(12)	0.4205(3)	0.030(3)
H21A	0.71126	0.75105	0.43520	0.044
H21B	0.66011	0.90381	0.42527	0.044
O22	0.82889(12)	0.2247(12)	0.3759(3)	0.035(3)
H22A	0.83970	0.14344	0.30690	0.053
H22B	0.78735	0.22837	0.35584	0.053
O23	0.57737(13)	0.7553(12)	0.5912(3)	0.036(3)
H23A	0.54562	0.70934	0.58707	0.055
H23B	0.59856	0.66035	0.60506	0.055
O24	0.66992(14)	0.5408(14)	0.6846(3)	0.040(4)
H24A	0.65666	0.60022	0.61139	0.059
H24B	0.70286	0.59088	0.70614	0.059
C3	.79488(13)	0.7641(12)	0.8277(3)	0.014(3)
C4	.70475(13)	0.2761(13)	0.4411(3)	0.025(3)
H13	.897(5)	0.157(10)	0.867(7)	0.08(3)

U_{eq} is defined as 1/3 of the trace of the orthogonalized U_{ij} tensor.

Table S2. Anisotropic displacement parameters [$Å^2$] for the HP1 phase.
The anisotropic displacement factor exponent takes the form:
$-2\pi^2[\ h^2(a^*)^2U_{11} + k^2(b^*)^2U_{22} + \ldots + 2hka^*b^*U_{12}\ ]$

Atom	U_{11}	U_{22}	U_{33}	U_{23}	U_{13}	U_{12}
Mg1	0.0156(5)	0.013(4)	0.0169(5)	−0.0016(7)	0.0029(3)	0.0006(8)
Mg2	0.0146(5)	0.022(4)	0.0166(5)	−0.0008(8)	0.0029(3)	0.0017(8)
Mg3	0.0160(5)	0.017(4)	0.0180(5)	−0.0005(8)	0.0030(3)	0.0009(8)
Mg4	0.0149(5)	0.014(4)	0.0171(5)	−0.0016(8)	0.0024(3)	-0.0001(7)

Table S3. Comparison of Experimental and Theoretical Hydrogen Bonds in Nesquehonite.

	Pressure (GPa)	H···O (Å)		∠DHA Angle (°)	
**Nesquehonite**	0.001	**H1 – O5**	1.95(4)	**O1 – H1 – O5**	161.4(17)
		**H2 – O6**	2.030(3)	**O1 – H2 – O6**	175(3)
		**H3 – O5**	1.90(4)	**O1 – H3 – O5**	162(3)
		**H4 – O6**	1.970(3)	**O1 – H4 – O6**	177(3)
		**H6 – O3**	1.93(3)	**O1 – H6 – O3**	174.4(17)
**Nesquehonite (DFT)**	0.2460	**H1 – O5**	1.734	**O1 – H1 – O6**	158.0
		**H2 – O6**	1.665	**O1 – H2 – O6**	174.4
		**H3 – O5**	1.725	**O2 – H3 – O5**	169.2
		**H4 – O6**	1.788	**O2 – H4 – O6**	167.8
		**H6 – O3**	1.678	**O6 – H6 – O3**	172.1

Table S4. Comparison of Experimental and Theoretical Hydrogen Bonds in HP1 phase.

	Pressure (GPa)	H···O (Å)		∠DHA Angle (°)	
**HP1 Phase**	3.1	**H13 – O22**	1.920(12)	**O13 – H13 – O22**	144(10)
		**H15A – O10**	1.940(4)	**O15 – H15A – O10**	136.4(3)
		**H15B – O19**	1.939(6)	**O15 – H15B – O19**	153.4(6)
		**H16 – O3**	1.936(5)	**O16 – H16 – O3**	142.3(5)
		**HA – O21**	1.930(5)	**O16 – HA – O21**	158.2(5)
		**H17 – O21**	2.078(6)	**O17 – H9 – O21**	131.4(4)
		**H18 – O23**	2.040(7)	**O18 – H18 – O23**	140.7(6)
		**HC – O11**	1.854(5)	**O18 – HC – O11**	166.4(4)
		**H19B – O13**	2.068(5)	**O19 – H19B – O13**	159.6(4)
		**H20A – O19**	1.740(5)	**O20 – H20A – O19**	178(6)
		**H21A – O7**	1.740(3)	**O21 – H21A – O7**	169(4)
		**H22A – O15**	1.903(5)	**O22 – H22A – O15**	154.1(4)
		**H22B – O5**	1.655(3)	**O22 – H22B – O5**	169.7(6)
		**H23A – O9**	1.896(3)	**O23 – H23A – O9**	152.3(5)
		**H23B – O24**	1.998(6)	**O23 – H23B – O24**	151.6(5)
		**H24B – O3**	1.809(5)	**O24 – H24B – O3**	163.1(6)
**HP1 Phase (DFT)**	3.5415	**H1 – O22**	1.717	**O13 – H1 – O22**	156.7
		**H5 – O10**	1.650	**O15 – H5 – O10**	162.7
		**H6 – O19**	1.713	**O15 – H6 – O19**	166.8
		**H7 – O3**	1.732	**O16 – H7 – O3**	159.3
		**H8 – O21**	1.685	**O16 – H8 – O21**	168.6
		**H9 – O21**	1.685	**O17 – H9 – O21**	161.1
		**H10 – O11**	1.718	**O17 – H10 – O11**	169.5
		**H11 – O23**	1.794	**O18 – H11 – O23**	162.0
		**H15 – O19**	1.657	**O20 – H15 – O19**	166.9
		**H17 – O24**	1.772	**O21 – H17 – O24**	156.0
		**H18 – O7**	1.644	**O21 – H18 – O7**	168.6
		**H20 – O5**	1.599	**O22 – H20 -O5**	173.2
		**H22 – O11**	1.772	**O23 – H22 – O11**	170.3
		**H23 – O3**	1.597	**O24 – H23 – O3**	173.3

Table S5. Atomic coordinates and U_{eq} [Å^2] for the HP2 phase

**Atom**	***x***	***y***	***z***	U_{eq}
Mg1	0.36730(10)	0.4603(4)	0.40409(9)	0.0131(10)
O3	0.3788(3)	0.4967(9)	0.5688(2)	0.017(3)
O1	0.21208(19)	0.4554(8)	0.27111(19)	0.016(2)
O2	0.49395(17)	0.4236(8)	0.27365(18)	0.0129(18)
O1W	0.37420(19)	0.1770(9)	0.42844(18)	0.0140(18)
C1	0.1095(2)	0.5080(10)	0.3246(2)	0.018(3)
O3W	0.35675(19)	0.9256(9)	0.59505(19)	0.0125(19)
O2W	0.3832(2)	0.7506(9)	0.39186(19)	0.0148(19)
H3WA	0.398(5)	0.970(13)	0.662(4)	0.018
H2WA	0.429(4)	0.833(15)	0.338(4)	0.018
H2WB	0.309(4)	0.839(14)	0.386(4)	0.018
H3WB	0.421(4)	0.842(14)	0.563(4)	0.018
H1WA	0.356(4)	0.850(12)	0.485(4)	0.018
H1WB	0.333(4)	0.112(15)	0.369(4)	0.018

U_{eq} is defined as 1/3 of the trace of the orthogonalized U_{ij} tensor.

Table S6. Anisotropic displacement parameters [Å^2] for the HP2 phase.

The anisotropic displacement factor exponent takes the form:
$-2\pi^2[\ h^2(a^*)^2U_{11} + k^2(b^*)^2U_{22} + \ldots + 2hka^*b^*U_{12}\ ]$

**Atom**	U_{11}	U_{22}	U_{33}	U_{23}	U_{13}	U_{12}
Mg1	0.0162(4)	0.012(3)	0.0114(4)	0.0007(6)	0.0014(3)	0.0008(5)
O3	0.0388(12)	0.002(9)	0.0117(9)	-0.0001(14)	0.0025(7)	0.0009(14)
O1	0.0112(7)	0.021(6)	0.0169(8)	0.0002(13)	0.0034(5)	0.0011(10)
O2	0.0105(7)	0.011(6)	0.0176(8)	-0.0021(13)	0.0010(4)	-0.0014(10)
O1W	0.0182(8)	0.009(6)	0.0145(8)	0.0009(13)	0.0019(5)	-0.0014(12)
C1	0.0111(8)	0.031(8)	0.0115(9)	-0.0004(14)	0.0014(5)	-0.0029(11)
O3W	0.0140(7)	0.006(6)	0.0174(8)	-0.0004(13)	0.0015(5)	0.0012(10)
O2W	0.0177(8)	0.007(6)	0.0197(9)	0.0015(15)	0.0041(5)	0.0006(11)

Table S7. Comparison of Experimental and Theoretical Hydrogen Bonds in HP2 phase.

	Pressure (GPa)	H···O (Å)		∠DHA Angle (°)	
		**H1WB – O1**	1.73(7)	**OW1 – H1WB – O1**	165(7)
		**H2WA – O2**	1.72(6)	**OW2 – H2WA – O2**	168(8)
**HP2 Phase**	11.6	**H2WB – O3W**	1.720(5)	**OW2 – H2WB – O3W**	157(7)
		**H3WA – O2**	1.70(6)	**OW3 – H3WA – O2**	171(7)
		**H3WB – O1W**	1.94(4)	**OW3 – H3WB – O1W**	137(6)
		**H6 – O2**	1.569	**O4 – H6 – O2**	166.7
		**H1 – O3**	1.592	**O5 – H1 – O3**	170.2
**HP2 Phase (DFT)**	11.7435	**H4 – O4**	1.710	**O5 – H4 – O4**	150.3
		**H2 – O3**	1.680	**O6 – H2 – O3**	175.7
		**H3 – O5**	1.576	**O6 – H3 – O5**	174.4

Table S8. Experimental Raman Modes to Nesquehonite under ambient conditions.

**Raman Shift (cm^{-1})**	**Raman Mode Assignment**
50.12	T Modes
92.49	
105.92	
116.71	
148.40	
165.31	
183.01	
193.59	
225.65	
272.97	L Modes
307.92	
343.46	
696.91	In-plane bending (ν_4)
761.47	
1098.45	Symmetric Stretching (ν_1)
1430.32	Antisymmetric Stretching (ν_3)
1513.65	

Figure S1. Coordination details of $[CO_3]$ carbonate groups in the nesquehonite structure: neighboring $[MgO_6]$ octahedra and hydrogen bonding.

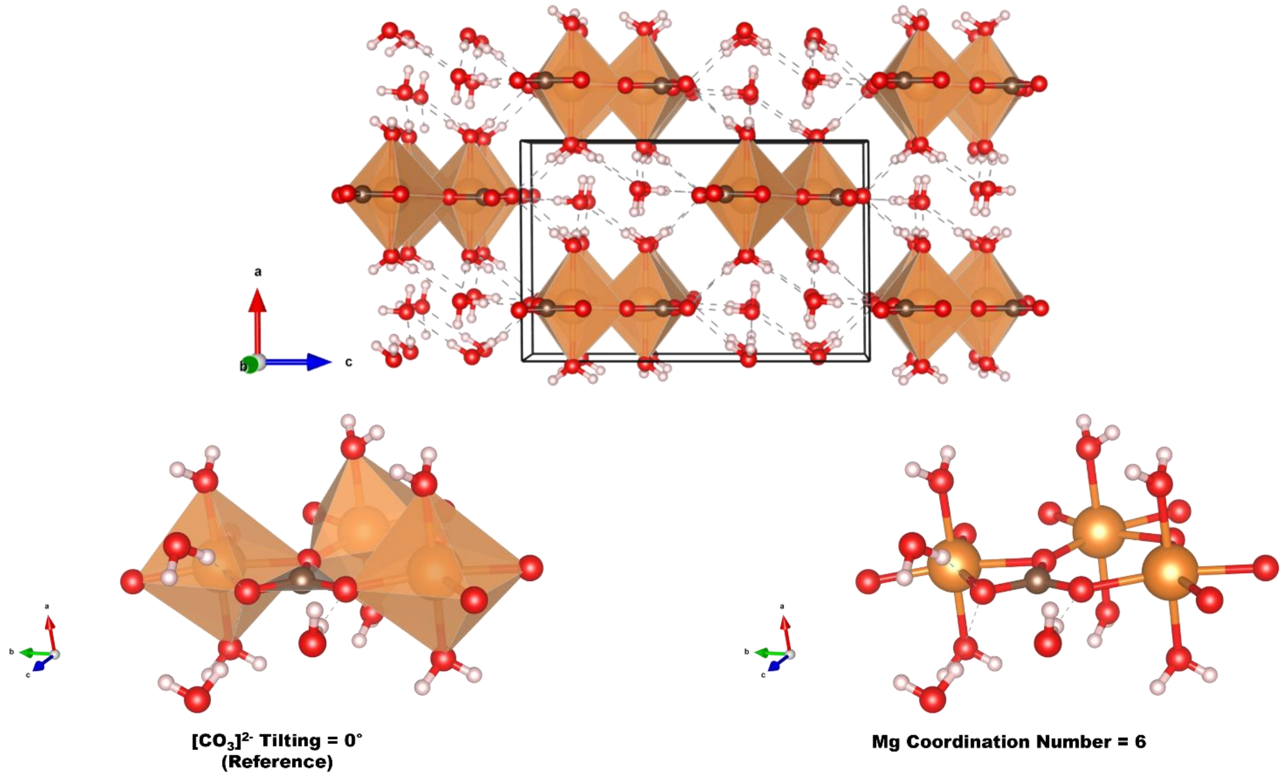

Figure S2. Coordination details of [CO_3] carbonate groups in the previously reported pr-HP1 phase[25]: neighboring [MgO_6] octahedra and hydrogen bonding.

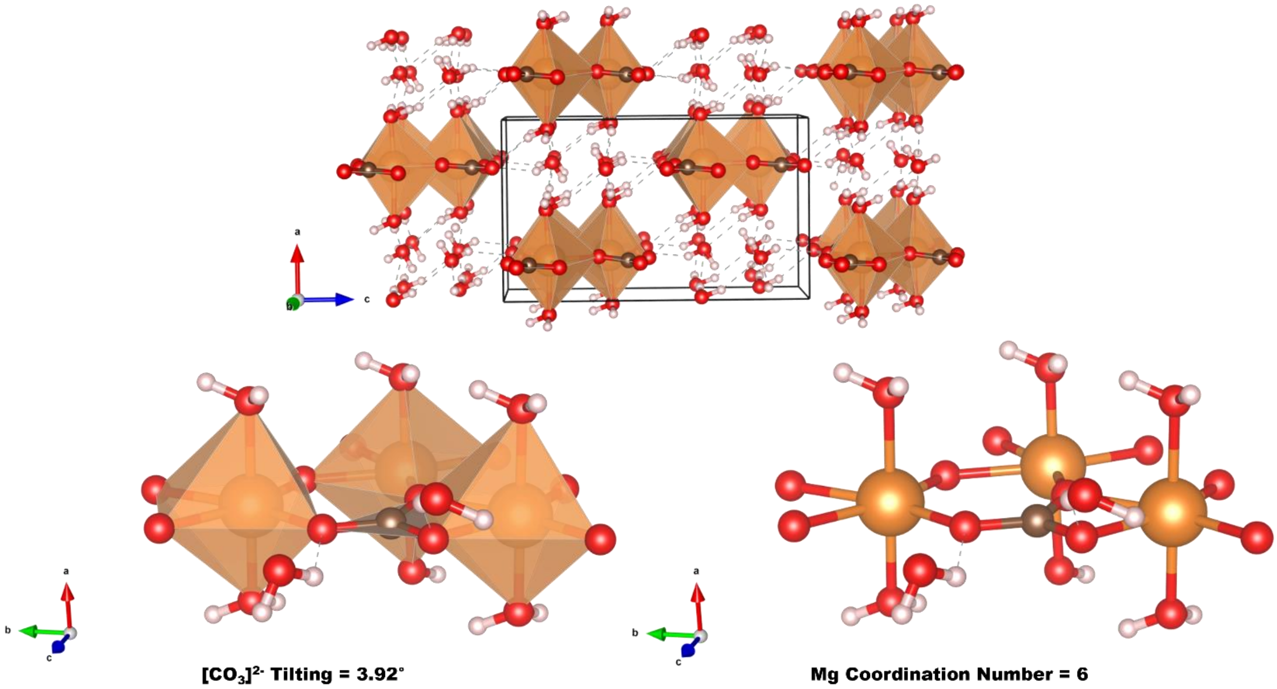

Figure S3. Coordination details of [CO_3] carbonate groups in the HP1 phase: neighboring [MgO_6] octahedra and hydrogen bonding.

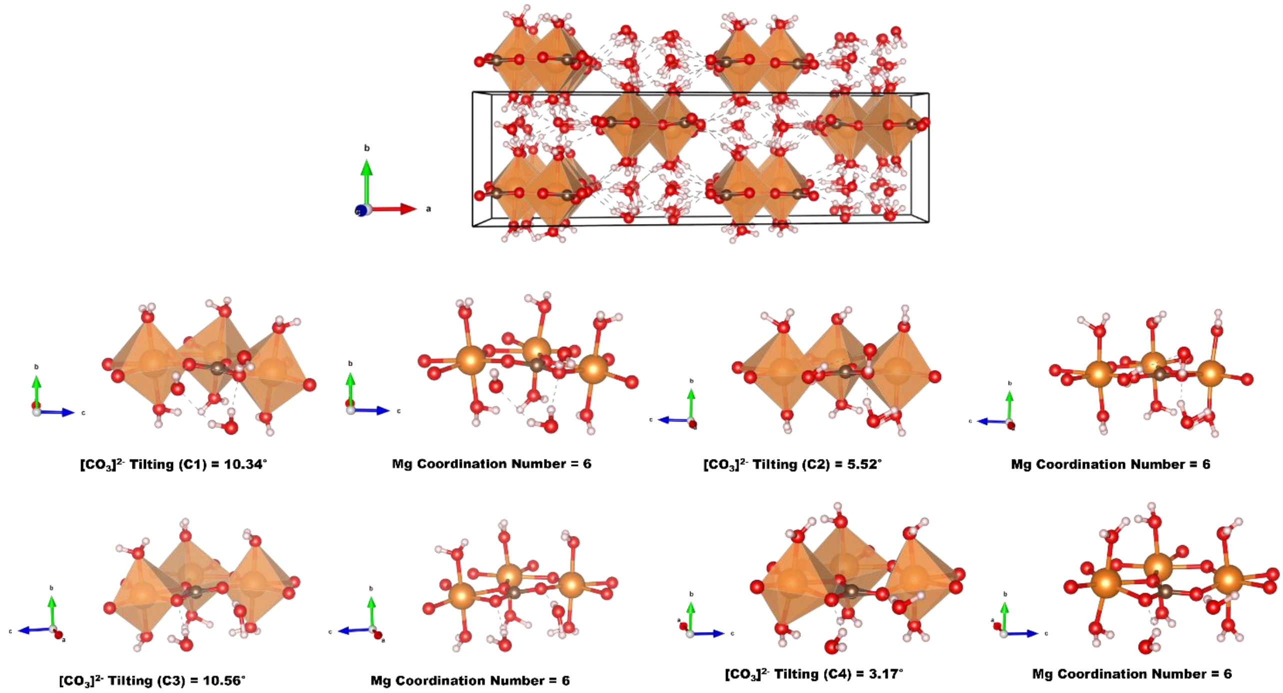

Figure S4.- LeBail fit of the integrated powder XRD pattern of $MgCO_3{\cdot}3H_2O$ at 2.75 GPa and room temperature using Ne as pressure transmitting medium using the P21/c unitcell of the HP1 phase. Observed, calculated and difference X-ray diffraction profiles are depicted in black, red, and green, respectively. Magenta vertical marks indicate Bragg reflections. Synchrotron radiation wavelength = 0.4246 Å.

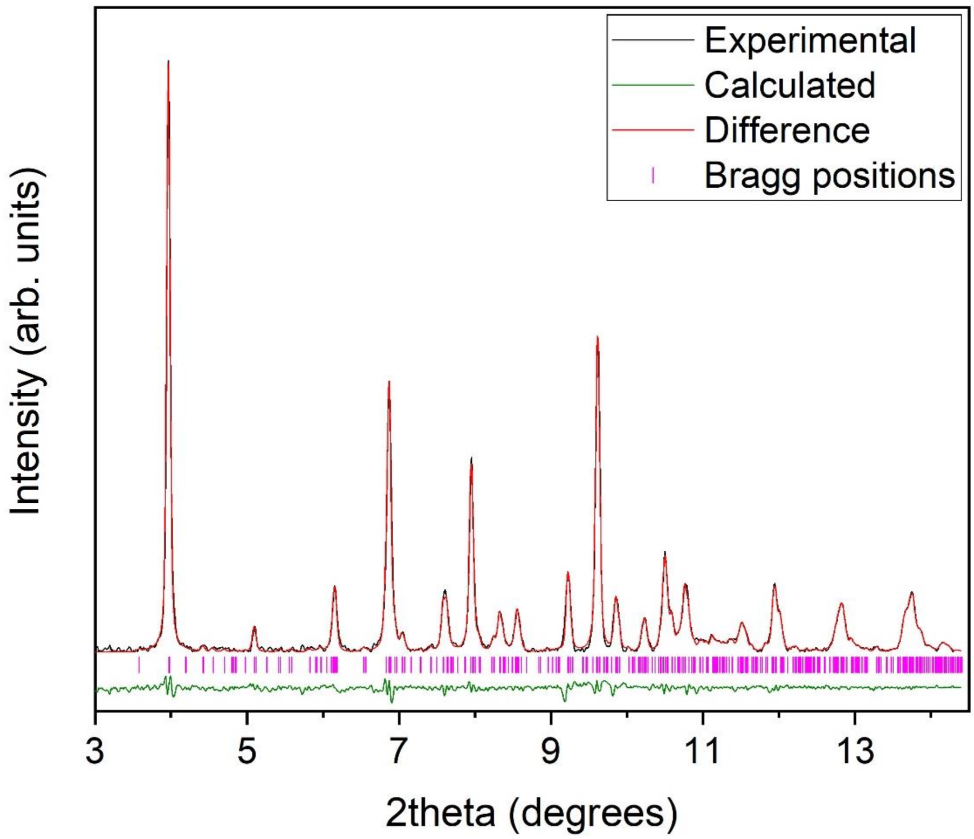

Figure S5.- Pressure dependence of the volume per formula unit of $MgCO_3{\cdot}3H_2O$ up to 19 GPa using the powder XRD data of Reference 25 and the structural models reported in this study. Solid symbols correspond to experimental data. The color codes given in the figure inset indicate the phase Magenta and green lines correspond to the experimental and DFT-calculated third-order Birch–Murnaghan equations of state.

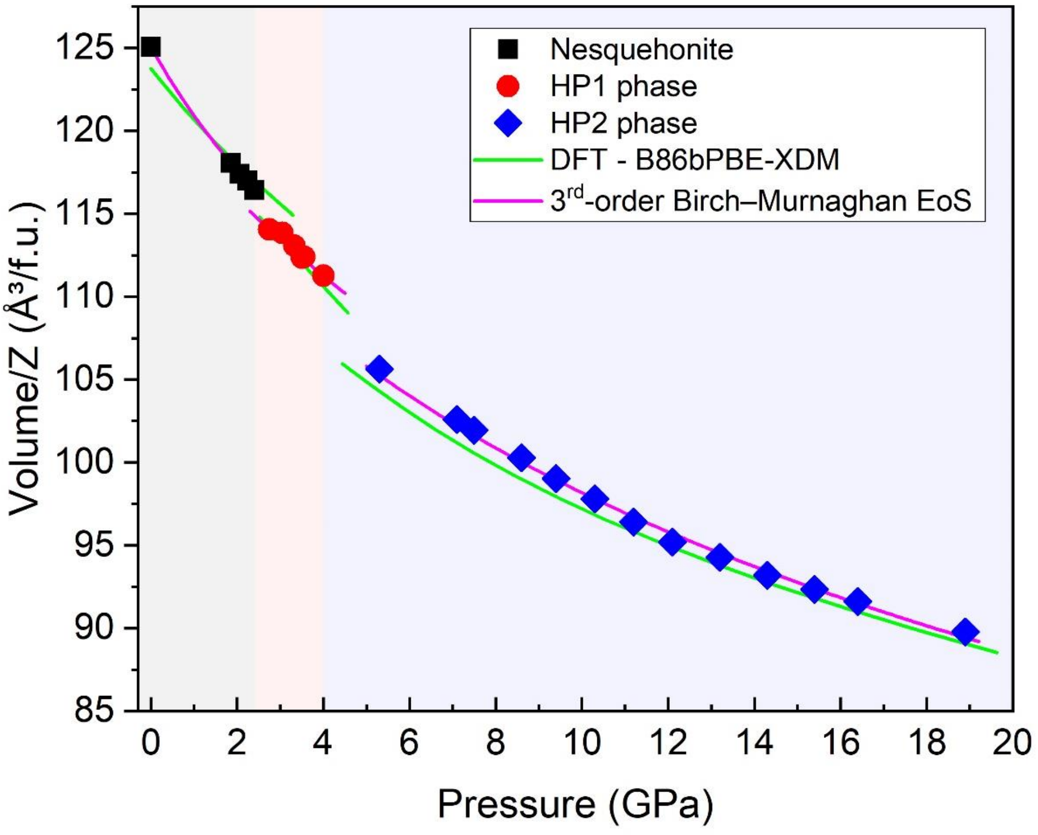

Figure S6. Scheme of a hydrogen bond, illustrating the bond length and angle collected in Tables 3, S3, S4 and S7.

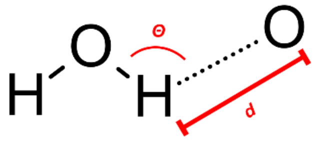

Figure S7. Coordination details of [CO_3] carbonate groups in the HP2 structure: neighboring [MgO_7] polyhedra and hydrogen bonding.

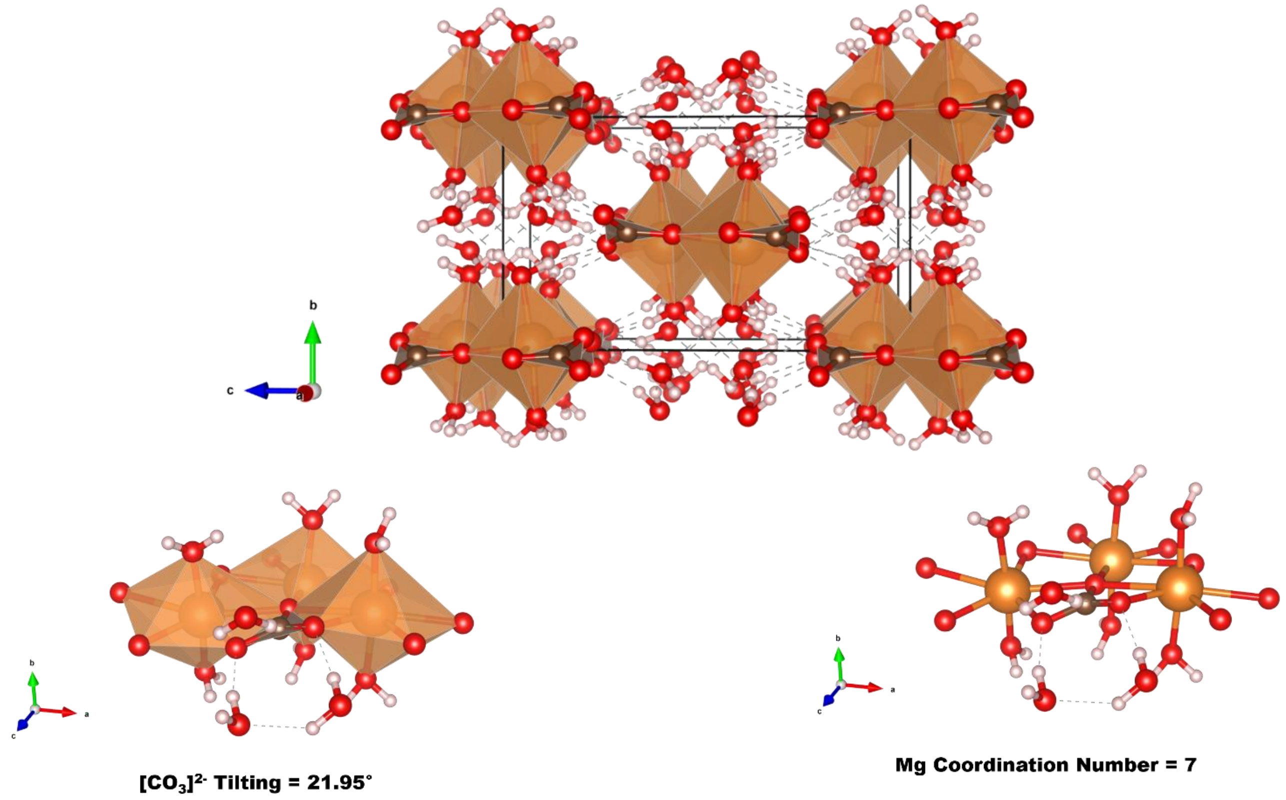

Figure S8- Plot of the enthalpy difference versus pressure for the combination $MgO + H_2CO_3 + 2 H_2O$ with respect to the most stable phase of $MgCO_3 \cdot 3H_2O$ nesquehonite at each pressure.

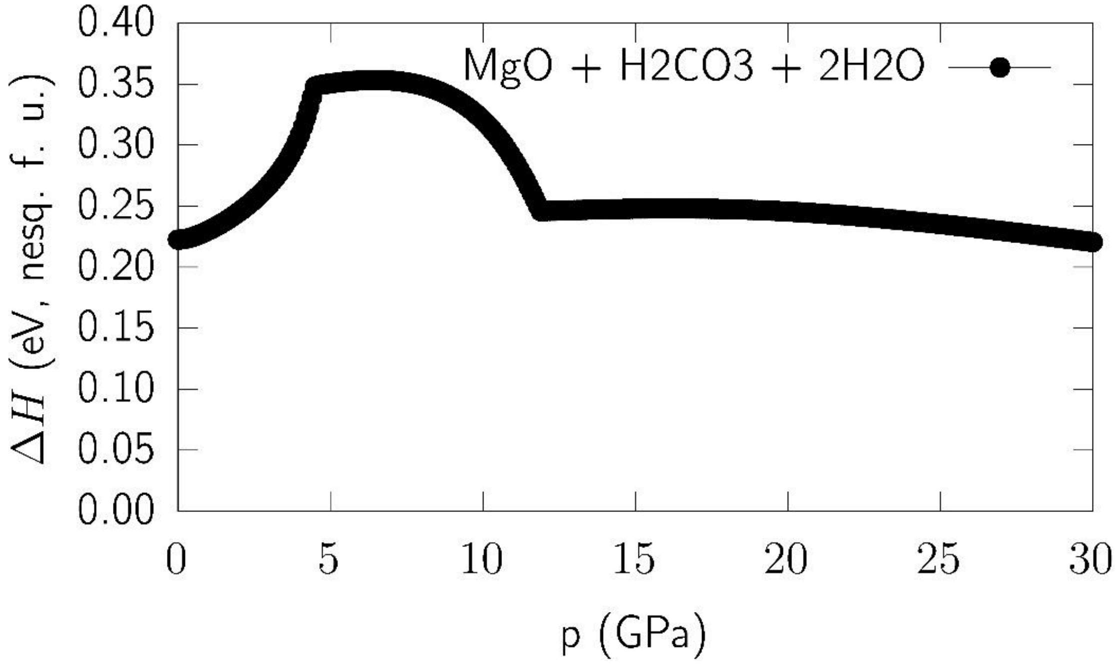

Figure S9.- LeBail fit of the integrated XRD pattern of $MgCO_3·3H2O$ at 5.3 GPa and room temperature using Ne as pressure transmitting medium. Observed and calculated, X-ray diffraction profiles are depicted in black and red, respectively. Magenta vertical marks indicate Bragg reflections. The cake image of the raw data is shown below. Synchrotron radiation wavelength = 0.4246 Å.

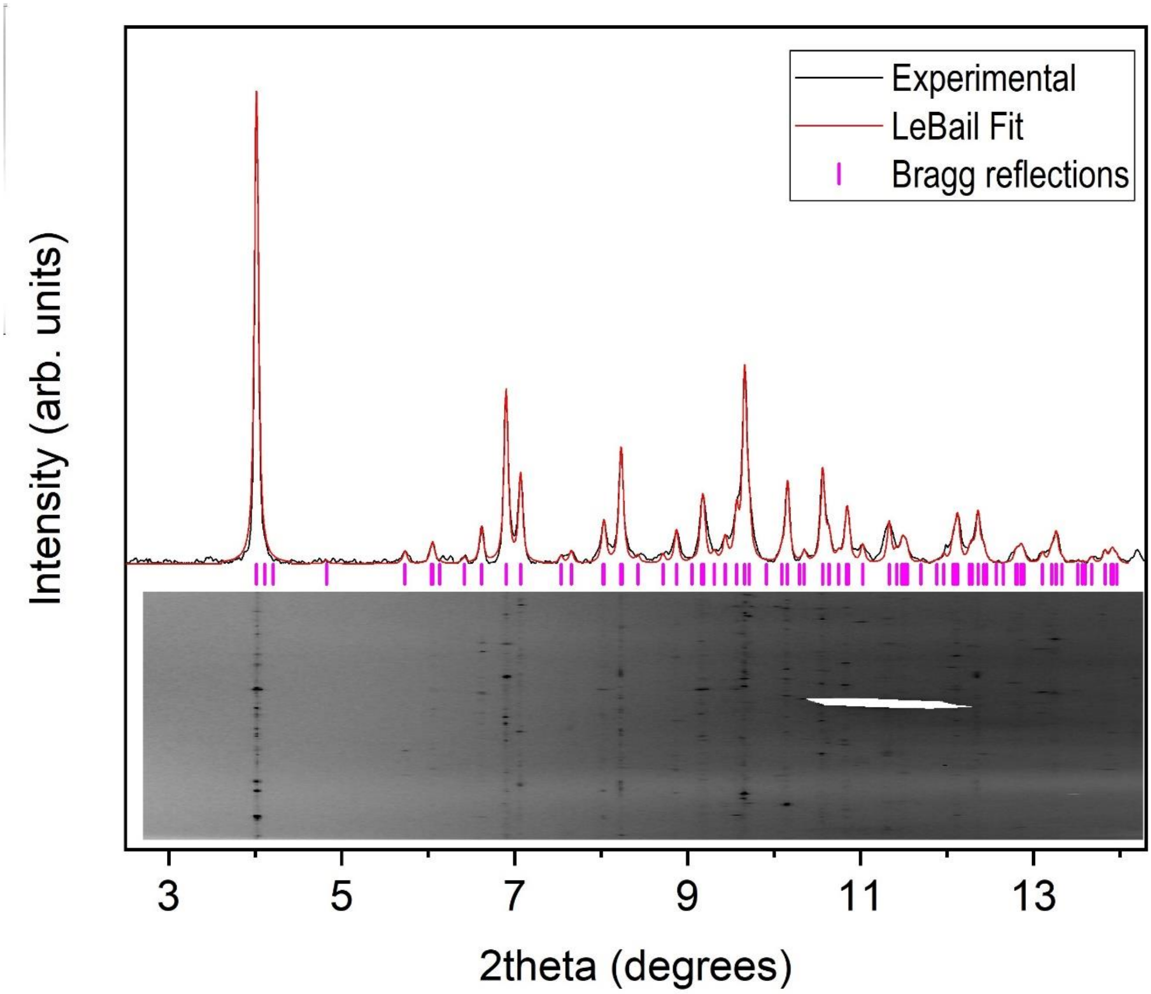

Supplement: Supplementary file 1 — ic4c01699_si_001.pdf [file ic4c01699_si_001.pdf]
